# Supplementary material for: A risk score model with five long non-coding RNAs for predicting prognosis in gastric cancer: an integrated analysis combining TCGA and GEO datasets
Source: PeerJ. 2021 Feb 9;9:e10556. doi: 10.7717/peerj.10556 (PMC7879943; doi:10.7717/peerj.10556)
Supplement: Supplemental Information 1 [file peerj-09-10556-s001.pdf]

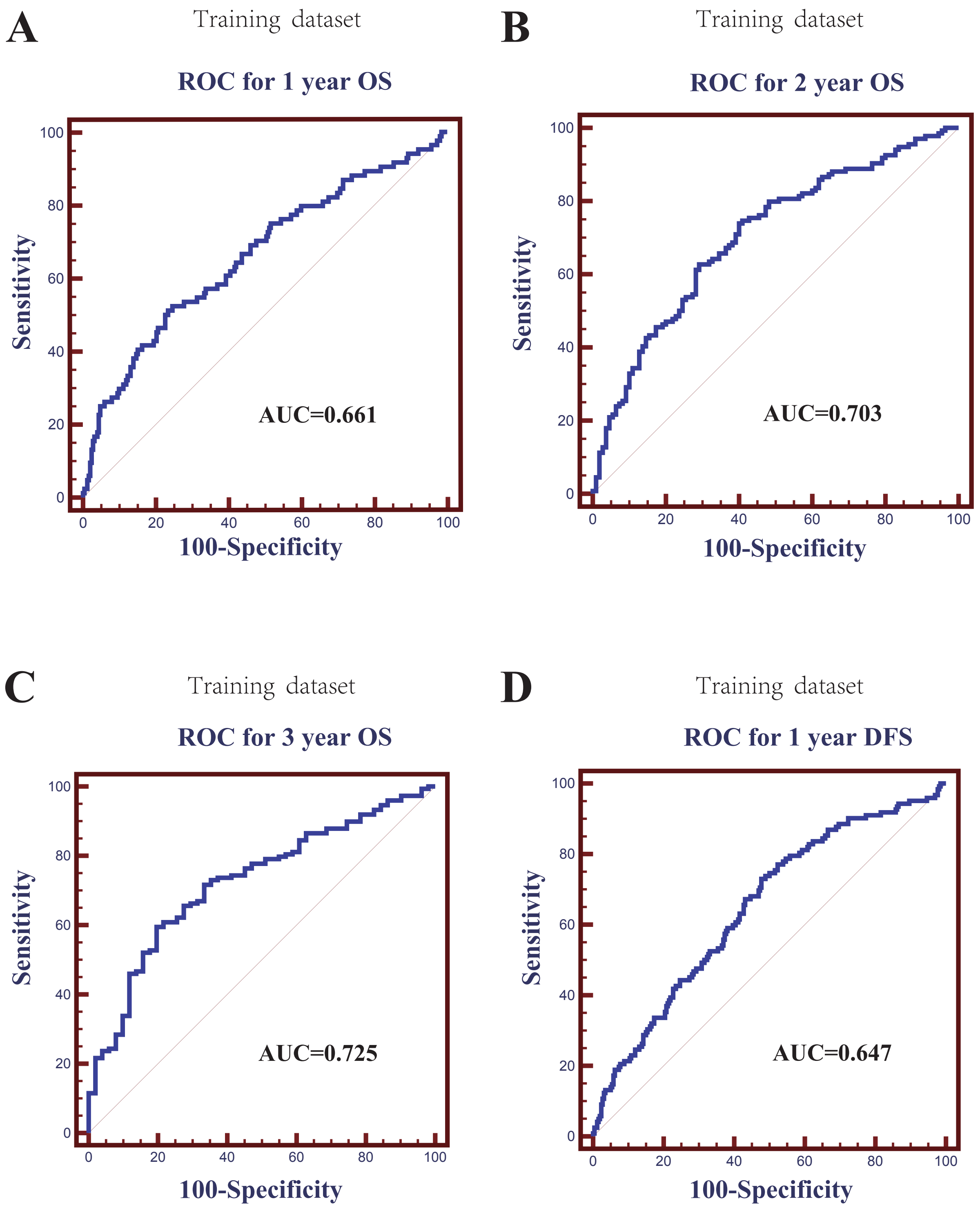
A
Training dataset
ROC for 1 year OS
AUC=0.661
Sensitivity
100-Specificity
0
20
40
60
80
100
B
Training dataset
ROC for 2 year OS
AUC=0.703
Sensitivity
100-Specificity
C
Training dataset
ROC for 3 year OS
AUC=0.725
Sensitivity
100-Specificity
D
Training dataset
ROC for 1 year DFS
AUC=0.647
Sensitivity
100-Specificity
