# Supplementary figures and images for: A risk score model with five long non-coding RNAs for predicting prognosis in gastric cancer: an integrated analysis combining TCGA and GEO datasets
Source: PeerJ. 2021 Feb 9;9:e10556. doi: 10.7717/peerj.10556 (PMC7879943; doi:10.7717/peerj.10556)

A

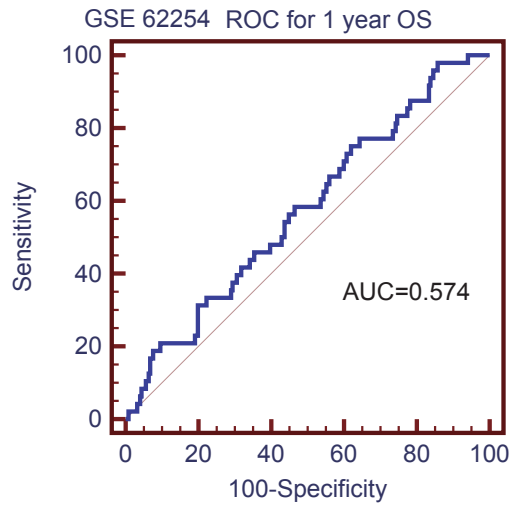

B

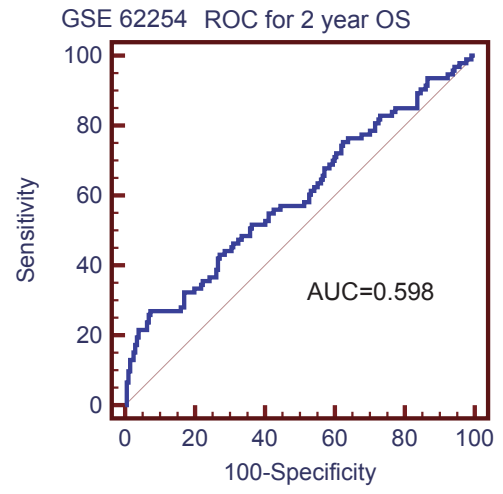

C

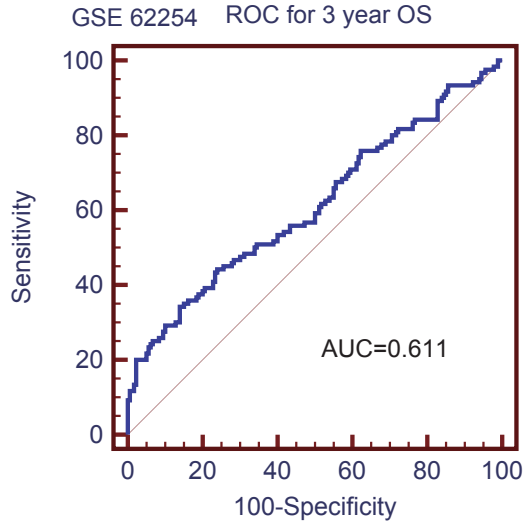

D

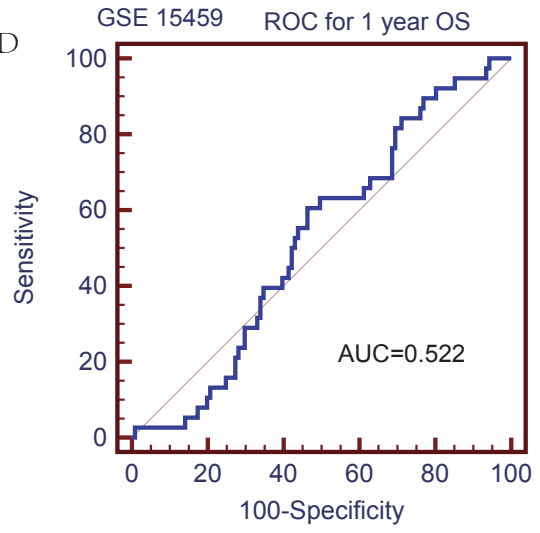

E

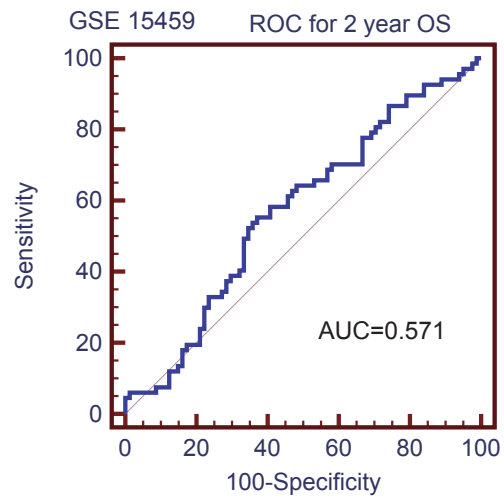

F

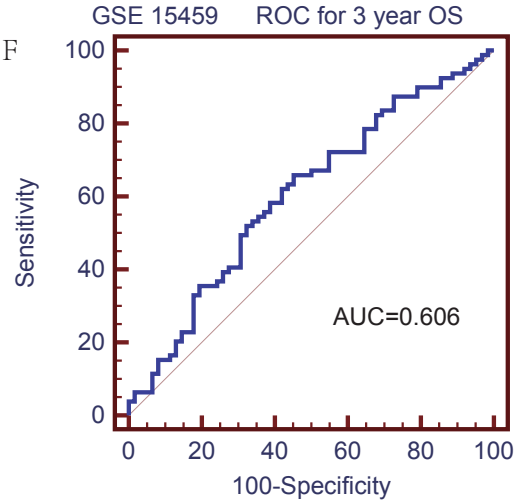

Supplement: Supplemental Information 2 [file peerj-09-10556-s002.pdf]

A

GSE 62254

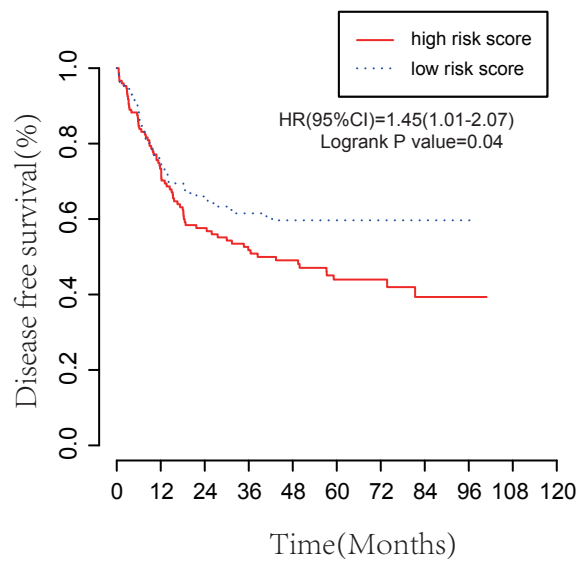

B

GSE 62254

■ Recurrence  
■ No Recurrence

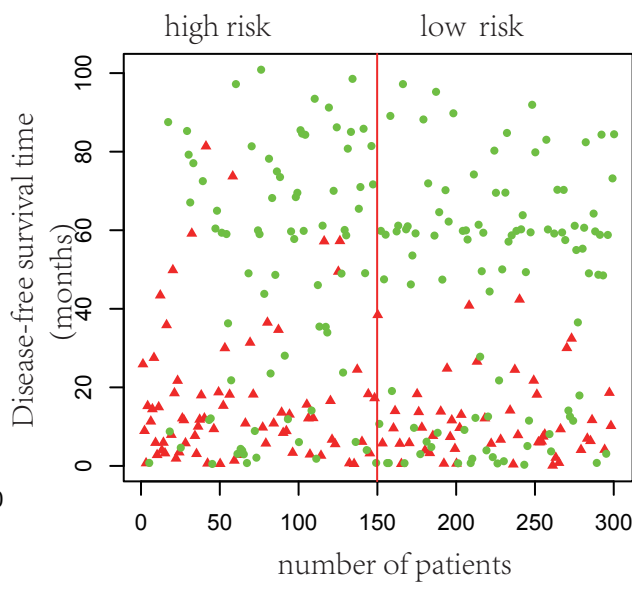

C

GSE 62254

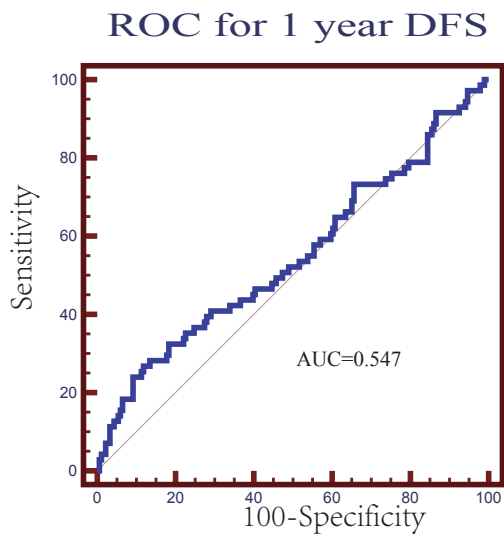

D

GSE 62254

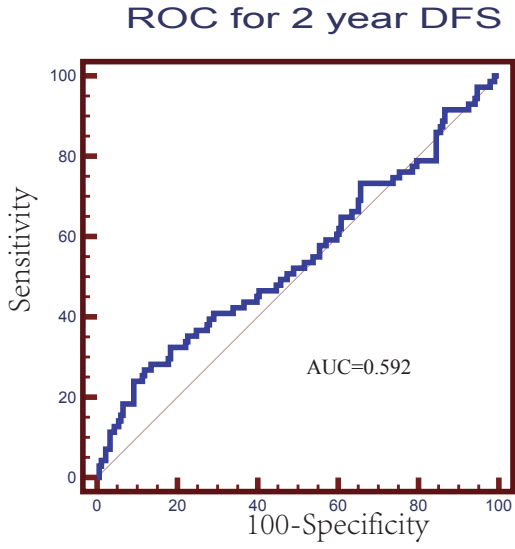

Supplement: Supplemental Information 3 — (A) Kaplan–Meier analysis of predicting DFS of GC patients based on the high risk and low risk subgroups in validation group (GSE62254). (B) The lncRNA-based model distribution for patient recurrence in validation group (GSE62254). (C–D) The time-independent ROC analysis of the risk score for prediction the 1-2 year cutoff DFS in validation group (GSE62254). The area under the curve was calculated for ROC curve. [file peerj-09-10556-s003.pdf]
